# Aspherical scattering factors for *SHELXL* – model, implementation and application

**DOI:** 10.1107/S2053273318013840

**Published:** 2019-01-01

**Authors:** Jens Lübben, Claudia M. Wandtke, Christian B. Hübschle, Michael Ruf, George M. Sheldrick, Birger Dittrich

**Affiliations:** a Institut für Anorganische Chemie der Universität Göttingen, Tammannstrasse 4, Göttingen, D-37077, Germany; b Bruker AXS Inc., 5465 E. Cheryl Parkway, Madison, WI 53711, USA; c Laboratory of Crystallography, University of Bayreuth, D-95440 Bayreuth, Germany; d Heinrich-Heine Universität Düsseldorf, Institut für Anorganische Chemie und Strukturchemie, Material- und Strukturforschung, Gebäude: 26.42, Universitätsstrasse 1, 40225 Düsseldorf, Germany

**Keywords:** *SHELXL*, invarioms, aspherical scattering factors, quantum crystallography

## Abstract

A new aspherical scattering factor formalism has been implemented in the crystallographic least-squares refinement program *SHELXL*. The formalism relies on Gaussian functions and can optionally complement the independent atom model to take into account the deformation of electron-density distribution due to chemical bonding and lone pairs. Asphericity contributions were derived from the electron density obtained from quantum-chemical density functional theory computations of suitable model compounds that contain particular chemical environments, as defined by the invariom formalism. Thanks to a new algorithm, invariom assignment for refinement in *SHELXL* is automated. A suitable parameterization for each chemical environment within the new model was achieved by metaheuristics. Figures of merit, precision and accuracy of crystallographic least-squares refinements improve significantly upon using the new model.

## Abstract 2

A new aspherical scattering factor formalism was implemented in *SHELXL*. It relies on Gaussian functions and can optionally complement the independent atom model to take into account the deformation of electron-density distribution due to chemical bonding and lone pairs. The automated atom-type assignment was derived from the invariom formalism.

## 1. Introduction   

According to a recent search in the Cambridge Structural Database (CSD) (Groom *et al.*, 2016 ▸) *SHELXL* (Sheldrick, 2015 ▸) is by far the most widely used computer application for crystallographic least-squares refinement (Cruickshank, 1970 ▸; Rollett, 1970 ▸) in small-molecule crystallography. In macromolecular crystallography (Sheldrick & Schneider, 1997 ▸) it is mostly used for structures with atomic resolution (better than 1.2 Å) (Sheldrick, 1990 ▸), especially when standard deviations of the refined bond distances and angles are being sought (Dauter, 2003 ▸) and when atomic displacement parameters (ADPs) (Trueblood *et al.*, 1996 ▸) can be refined. *SHELXL* has seen continuous development and improvements since about 1970, but the first official release was 1976 (Sheldrick, 2008 ▸). The implementation of new features often followed research by other scientists after algorithms and concepts reached a certain level of maturity and showed their value. This is exemplified by the choice of refinement against intensities and not structure factors as a default (Hirshfeld & Rabinovich, 1973 ▸; Arnberg *et al.*, 1979 ▸). In other cases genuinely new ideas and features were implemented in the program, as for instance in the new RIGU restraints (Thorn *et al.*, 2012 ▸). In these code modifications and extensions, the emphasis has always been on robustness and reliability, user friendliness, conceptual elegance (≃ simplicity), compatibility with previous versions and speed. We think it is this philosophy, as well as some early design decisions, including the choice of the programming language Fortran, that led to the success of *SHELXL*.

*SHELXL* implements the independent atom model (IAM) (Doyle & Turner, 1968 ▸). In this simple and elegant model each element in the periodic table has a single corresponding uncharged scattering factor, even when atoms are involved in chemical bonding and thus are not strictly identical. The analytical representation of the IAM scattering factor, which is the Fourier transform of an atomic electron density 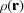, is usually incorporated as an overlay of four Gaussian functions,
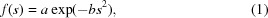
with element-specific coefficients 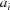 and exponents 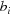 and optionally a constant *c*, as tabulated in the *International Tables for Crystallography*, Volume C (Prince, 2004 ▸),^1^

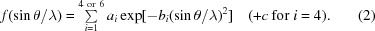
The main reason for using Gaussian functions for the IAM is the execution speed of analytical Fourier transform. However, their properties are not entirely satisfactory (Murshudov, 2016 ▸): firstly, form factors should be proportional to 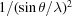 at high resolution, but Gaussian functions cannot mimic this proportionality; secondly, these functions are not ideally suited to model atomic charges to go beyond the IAM, since charge transfer mainly affects diffraction at low resolution.

Since its founding study (Coppens, 1967 ▸) charge-density research^2^ has shown that the experimental electron-density distribution 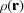 (EDD) deviates from its IAM representation in bonding and lone pair regions (Koritsánszky & Coppens, 2001 ▸), and this was predicted as early as 1915 (Debye, 1915 ▸). Today, with major improvements in detector technology and routine low-temperature measurements, such deformation density^3^ becomes visible ever more frequently in the residual electron density 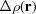. We believe that with this information becoming more routinely available, modern least-squares refinement programs should be able to model it. When this is achieved, structures become more precise as well as more accurate (Coppens *et al.*, 1969 ▸; Brock *et al.*, 1991 ▸; Jelsch *et al.*, 1998 ▸; Dittrich *et al.*, 2007 ▸). This article is about a new aspherical scattering factor model that was implemented in *SHELXL*.

## 2. Methods and models   

### 2.1. Scattering factor models in charge-density research and quantum crystallography   

Developments to improve scattering factors started in the early 1960s.^4^ The most established way to model aspherical EDD was conceived by Robert F. Stewart and is often called the rigid pseudoatom or multipole model (Stewart, 1976 ▸). An alternative earlier model with different angular functions but similar capabilities was developed by Hirshfeld (1971 ▸). Stewart’s approach has been modified by Hansen & Coppens (1978 ▸). Their multipole model (from now on called the SHC model) includes refinable radial screening parameters and has been widely used to ‘measure’ experimental EDD by least-squares refinement of aspherical population parameters normalized for electron density. The SHC model dominated charge-density research for decades, and contributed to answering numerous research questions at the interface between chemistry and physics (Koritsánszky & Coppens, 2001 ▸). Several computer programs have implemented the SHC model: *MOLLY* (Hansen, 1978 ▸), *VALRAY* (Stewart *et al.*, 1998 ▸), *MoPro* (Jelsch *et al.*, 2005 ▸), *XD2006* (Volkov *et al.*, 2006 ▸), *Jana2006* (Petříček *et al.*, 2014 ▸) and *WinXPRO* (Stash & Tsirelson, 2014 ▸).

More recently, it has become increasingly obvious that quantum-chemical computations in the framework of density functional theory (DFT) are powerful enough to faithfully reproduce molecular experimental EDD for light-atom structures.^5^ Therefore, the atom-centered SHC multipole model has more recently also been used to represent transferable EDD obtained from DFT computations. These efforts led to the construction of the invariom^6^ and other scattering factor databases.^7^ An alternative to tabulating scattering factors is Hirshfeld atom refinement (Jayatilaka & Dittrich, 2008 ▸; Capelli *et al.*, 2014 ▸), where a quantum-chemical mol­ecular EDD is partitioned to provide tailor-made aspherical atomic scattering factors for the molecule of interest. Here the philosophy is different than in classical charge-density research: the experimental results are atomic positions and ADPs,^8^ but not valence EDD anymore, since only positional and displacement parameters are refined. Both approaches lead to a considerably better agreement of 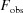 and 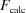, and more accurate and precise structures can be measured. This becomes most apparent for H atoms and their bond distances (Stewart *et al.*, 1975 ▸; Dittrich *et al.*, 2005 ▸; Woińska *et al.*, 2014 ▸; Dittrich *et al.*, 2017 ▸; Malaspina, Edwards *et al.*, 2017 ▸; Malaspina, White *et al.*, 2017 ▸).

Work by Dadda *et al.* (2012 ▸) and Nassour *et al.* (2014 ▸) deserves special emphasis, since some of the concepts used here are similar to their ‘virtual-atom’ refinement. Differences are technical in nature, but nevertheless important for everyday use. While these authors maintain a separation of core and valence electron density in analogy to the SHC model, we retain the IAM and model additional deformation density. Both approaches share the motivation to better model conventional data sets or those measured for macromolecular crystals with limited scattering power.

### 2.2. The bond-oriented deformation density (BODD) model in perspective   

A new Gaussian-based scattering factor model is being introduced here. It is fully integrated into a well established refinement program and is therefore more widely applicable than previous scattering factor databases and Hirshfeld atom refinement alike. For instance, the BODD model can be used to model twinned (Herbst-Irmer & Sheldrick, 1998 ▸) as well as disordered (Müller *et al.*, 2006 ▸) structures. The electron-density model described next is not intended to refine an experimental EDD, but was solely designed to model experimental data better on the basis of suitably partitioned (Dittrich *et al.*, 2004 ▸) theoretical computations of model compounds.

One important characteristic of the new model – in contrast to the SHC multipole model with its monopole populations and the ‘virtual-atom’ model – is that the scattering factors themselves do not carry a charge. Rather, the model constitutes a re-distribution of an atomic EDD into bonds and lone pairs for each individual atom. This is an important feature, because it allows the simultaneous combination of aspherical and conventional IAM scattering factors, *e.g.* to model compounds that also contain heavier elements. The validity and particular usefulness of this combination have been exemplified for coordination compounds (Wandtke *et al.*, 2017 ▸), albeit with a different refinement program and relying on the SHC model: the main challenge in the refinement of these metal-containing compounds with database parameters is that an exponentially increasing number of chemical environments would need to be tabulated for the central atoms.^9^ Coordination compounds are also amenable to Hirshfeld atom refinement. However, we then observe an exponential increase in computation time for compounds with electron-rich atoms due to the number of basis functions required. Both earlier approaches are hence unsuited for everyday use.

The EDD not described adequately by the IAM is on the one hand the EDD in bonding regions and on the other hand the EDD associated with lone pairs. Therefore, the concept of BODD is divided into two parts, one for modeling the bonding electron density (BEDE) and one for modeling the lone pair electron density (LONE). Both can be evoked by new commands in *SHELXL*.

### 2.3. Bonding electron density: BEDE   

**Figure 1:**
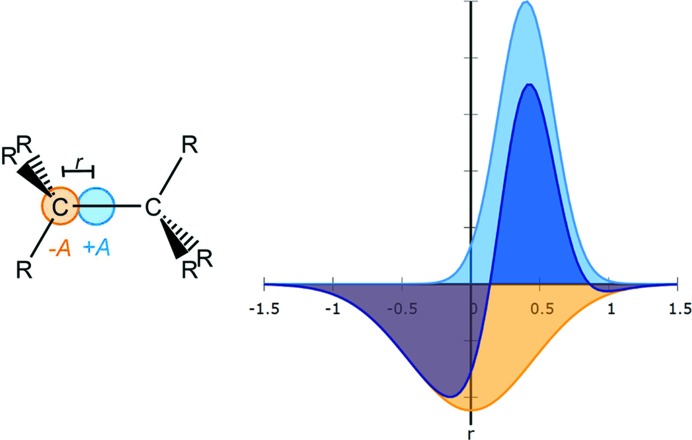
Left: basic concept of BEDE instruction for a standard covalent bond seen from the bonding direction going from atom1 to atom2. Right: generating asphericity from Gaussian functions while avoiding atomic charges.

The idea behind BEDE is based on density deformation functions similar to dipoles. They add electron density (ED) in the direction of the bond, and in order to keep the overall electron count correct, subtract ED at the atomic positions. If, for example, a covalent bond between two 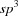 C atoms is described, the deformation will look as presented in Fig. 1 ▸. The correction is usually applied to a bond from both directions.

In the BEDE model Gaussian functions [see equation (1)] are positioned on the bond and at the atomic position, resulting in two Gaussians each with position *r*, amplitude *A* and spread *B*. This amounts to six parameters per bonding direction of an atom. Because EDD should not simply be added, the amplitude *A* at the atomic position of atom1 is the negative of the amplitude at the bond, reducing the number of parameters by one. The position of the Gaussian function is fixed with respect to the atomic coordinates, which leads to four parameters per bond per atom.

The instructions for *SHELXL* have the following syntax:

BEDE atom1 atom2 *r* *A* *B*1 *B*2 where: atom1 is the first atom involved in the bond, *r* is the distance between the Gaussian function and atom1 along the bond to atom2, and *A* is the amplitude for the Gaussian function on the bond. Likewise *A* is also the negative amplitude of the Gaussian function at atom1’s position, as visualized in the right part of Fig. 1 ▸. *B*1 is the spread of the Gaussian function with +*A* and *B*2 the spread of the Gaussian function with −*A*. Both *B* values are multiplied by the displacement parameters of atom1. Reference values for *A*, *B*1 and *B*2 are obtained by refining them as *SHELXL* free variables against intensity data generated by Fourier transformation of theoretical electron density. For structure refinement against experimental data they can be held fixed, but it turns out to be advantageous to refine up to three global scale factors for them.

### 2.4. Lone pair electron density: LONE   

**Figure 2:**
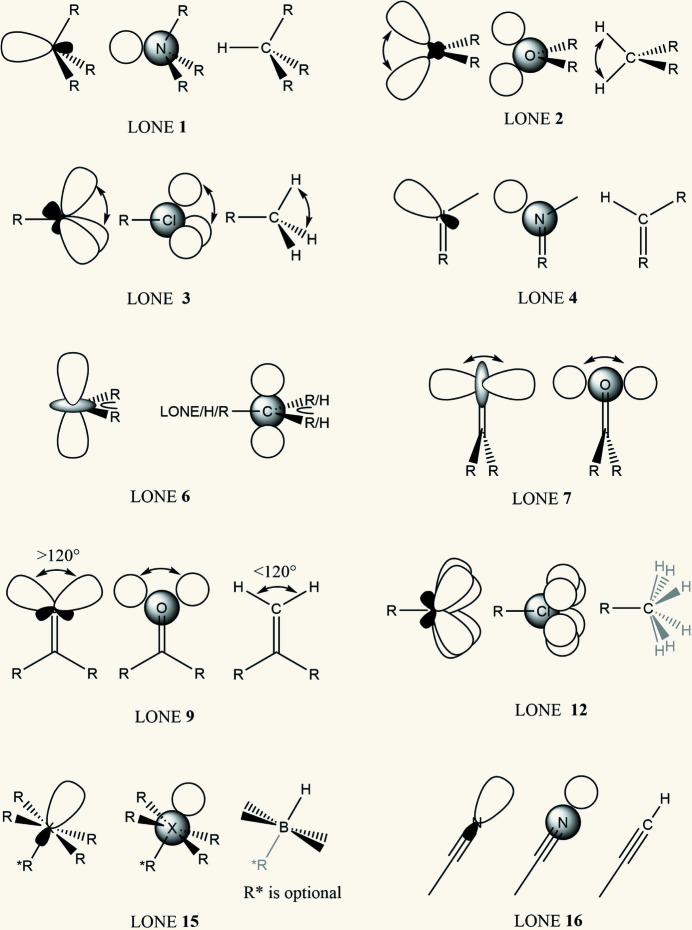
Concept and the different options *m* for the LONE instruction. The H-atom treatment corresponding to the same *m* is also shown, including angle expectations according to the VSEPR model.

The task of finding the direction for a function to model EDD in lone pairs is not as straightforward. Here a procedure analogous to deducing the orientation of H atoms from the other bonds connected to an atom is relied upon. *SHELXL* uses the number *m* to classify what kind of geometrical arrangement an atom is in. An overview of all possible positions for lone pairs and their H-atom analogs (where applicable) is presented in Fig. 2 ▸. The syntax thus includes *m* instead of a second atom, and in some cases an additional angle:

LONE *m* atom *A* *B*1 *B*2 *r* [angle]

The angle applies only to *m* = 2, 3, 7 and 9 and is in those cases fixed like it is for the H atoms. As known from the VSEPR theory (Gillespie, 1963 ▸, 1970 ▸) the angle may deviate from that of the ideal geometry due to valence shell repulsion.^10^ Similar to BEDE *B*2 is the coefficient in the exponent of the subtracted Gaussian function. In those cases where more than one Gaussian function with amplitude *A* is created, the Gaussian at the named atom will have an amplitude which is a multiple of *A*, so that the number of electrons stays balanced. For example, for *m* = 2 the subtraction at the atom will be of 2*A*. A special case is *m* = 12, which corresponds to a disordered methyl group with two half-occupied positions rotated from one another by 60°, thereby placing 12 half lone pairs in a circle, where the amplitude of the Gaussian function at the atom will be −6*A* with *A* being the height of each of the 12 Gaussian functions. For *m* = 6 and 7 there is no hydrogen analog, because these LONE instructions are meant to be applied to π-bonds. While *m* = 6 places Gaussian functions above and below the atom named in the direction perpendicular to the plane defined by two bonds (preferably to non-H atoms), *m* = 7 does the same for terminal atoms where in order to define a plane the second bond is connected to the neighboring atom. *m* = 9 is similar to *m* = 7 but places the two Gaussian functions in the plane of the atoms instead of perpendicular to it. The case of *m* = 15 can be used in cases of lone pair placement for atoms with four or five bonds and one lone pair, for example SF_4_ or BrF_5_. For the modeling of π-bonds a combination of several BEDE and LONE instructions is suggested, and there is a ‘LONE 6’ command for this purpose.^11^ In the case of a carbonyl bond there are no other bonds originating from the O atom, so the subtraction of ED comes from the direction of the C atom.

In summary, BEDE and LONE provide an additional deformation model on top of the IAM that seamlessly extends its capabilities. This in turn means full downward compatibility with earlier refinements that can still be performed exactly as before. We think that this is a requirement for a widely applicable approach, since for some applications and research questions the IAM remains entirely sufficient and appropriate. Another characteristic is that the BODD approach, as suggested by its full name ‘bond-oriented deformation density’, does not require a local atomic coordinate system. Every Gaussian function used is attached or directed to an atom that thereby becomes aspherical. A disadvantage in this context is that BEDE and LONE do not reach the same sophistication in representing electron density as invarioms, transferable aspherical atom models (TAAMs) or Hirshfeld atom refinement; fine features of the EDD are not as well represented in the scattering factor model.^12^ The goal of the new model is not to replace earlier charge-density or current quantum-crystallography (Massa *et al.*, 1995 ▸; Grabowsky *et al.*, 2017 ▸; Tsirelson, 2018 ▸) models, but to open up a still rather narrow research area to the wider crystallographic community. BODD can complement more sophisticated approaches in applications where conceptual simplicity, easy implementation and execution speed are important. Speed is why we chose Gaussian functions over Slater functions, as the former permit fast analytical Fourier transform.

### 2.5. Usage   

BEDE and LONE commands are fully compatible with all other features of *SHELXL*. The user first performs a conventional refinement. Subsequently the BEDE and LONE instruction commands are added. Currently this is done automatically by evoking the *APEX3* software.^13^ The *APEX3* graphical user interface now contains a Python plugin that assigns the asphericity parameters and allows manual modification by the user. Technically the BEDE and LONE parameters are contained in an external file, which is referred to in the INS file. After assignment the BODD refinement can be directly performed by a click of the mouse.

As pointed out earlier, BEDE and LONE are not meant for free refinement of asphericity parameters with experimental data. In contrast to the multipole model, which was designed for this purpose and where the functions were chosen to minimize parameter correlation, it is strictly not advised to freely refine BEDE and LONE parameters, as results cannot be expected to be chemically or physically meaningful. Rather, BEDE and LONE parameterization is done using DFT computations of suitable model compounds, for the choice of which we rely on the notation of the respective entries of the invariom database (Dittrich *et al.*, 2013 ▸). The best set of individual BEDE and LONE values are contained in a database and were obtained by metaheuristics; Appendix *A* [App appa] contains full details of the respective technical implementation. These parameters should therefore only be used as fixed additions to the IAM scattering factors. The philosophy is hence similar to invariom and Hirshfeld atom refinement.

## 3. Results   

### 3.1. Effects of BODD modeling on common quality indicators   

In this section the benefits of modeling aspherical EDD due to chemical bonds and lone pairs *via* the proposed method are investigated using a series of structure models taken from the literature. For this purpose model quality is assessed by the value of 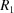 computed before and after applying the method. A drop of 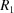 is expected after applying the BODD method. Since the absolute scale of the aspherical EDD parameters taken from the database is unknown in each particular structure, three scale factors were added in the refinements, multiplying all *A*, *B*1 and *B*2 parameters.

Table 1 ▸ summarizes the effects of modeling aspherical EDD for 15 structures of organic compounds.^14^ The table proves that the addition of aspherical EDD to the IAM structural model leads to a better fit to the data and most likely improved the physical significance of the structural models in all cases.

Since three scale factors were added to the structure model, the data-to-parameter ratio is reduced. To show that improvements in the fit are significant, a series of five structures was investigated in more detail in order to test whether the drop in 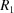 is caused by improving the model or by overfitting. This was done by computing 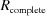 for all five structures before and after applying the BODD model (Lübben & Grüne, 2015 ▸). 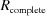 quantifies the amount of bias in the structural model. If the amount of bias is reduced after applying the BODD model, the BODD model does in fact improve the structural model significantly, and this is indeed the case here.

The bias *b* of a structural model can be quantified *via* 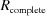 as follows: 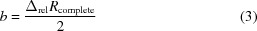


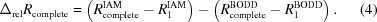
A positive value of *b* indicates a reduction of bias upon introducing the BODD model. A negative value indicates added bias.

Table 2 ▸ shows that application of the BODD model reduces the amount of bias, supporting the hypothesis that it improves the structural model by taking bond electron density into account.

Statistical analysis shows that the improvement in atomic positions for the non-H atoms is rather small (results not shown). This is similar to invariom refinement and Hirshfeld atom refinement. More interestingly, it is possible to freely refine meaningful H-atom positions with BODD with high-quality data. There have been detailed studies on this matter using neutron data as reference since the first study of this kind with theoretical aspherical scattering factors (Dittrich *et al.*, 2005 ▸) was performed, and similar results (albeit with slightly shorter average *X*⋯H distances than in Hirshfeld atom refinement or invariom refinement due to the use of only dipolar functions) can also be obtained with the BODD model. We decided not to report such results here, since our method should be generally applicable; when high-quality data are not available it is probably preferable to use AFIX constraints and elongate the *X*⋯H bond vector rather than to freely refine H-atom positions. We then suggest using an elongation factor of 1.14 for BODD. Moreover, the interested reader can easily perform such tests, since the model will be widely available.

While non-H-atom positions remain rather unaffected when including BODD contributions, including asphericity has a profound and systematic effect on the ADPs for both H and non-H atoms. Here inclusion of BEDE and LONE parameters leads to systematically larger H-atom 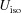
’s and smaller non-H-atom ADPs in better agreement with the reference invariom refinement, in analogy to earlier results for the multipole model (Jelsch *et al.*, 1998 ▸; Dittrich *et al.*, 2007 ▸). These findings are also in agreement with our earlier article (Lübben *et al.*, 2014 ▸) on H-atom ADPs. Using a temperature-dependent multiplier (Madsen & Hoser, 2015 ▸) ratio for the H-atom constraints (or free refinement of 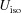) will thus be useful for BODD refinements with data measured at cryogenic temperatures. Another observation concerns the overall scale factor (OSF). As reported before, when applying theoretical aspherical multipole scattering factors (Volkov *et al.*, 2007 ▸), the OSF also usually becomes smaller (see Table 3 ▸). The situation is different for xylitol, where data were affected by extinction. The suggested weighting scheme values also become smaller overall; this also holds for the second value which up-weighs reflections similar to 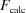. These findings can be complemented by statistical analysis. Given a set of *N* values 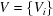 the mean value and its population standard deviation are defined by
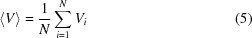


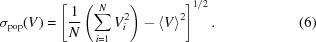
The population standard deviation 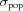 or root-mean-square deviation (RMSD) gives an indication of the spread of the values around the mean. To assess the similarity of individual ADP values we provide the mean difference MD (rather than the mean value) and its standard deviation 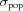 in Table 3 ▸. Subsequently the ADPs are also visually compared using *Peanut* plots (Hummel *et al.*, 1990 ▸).

**Figure 3:**
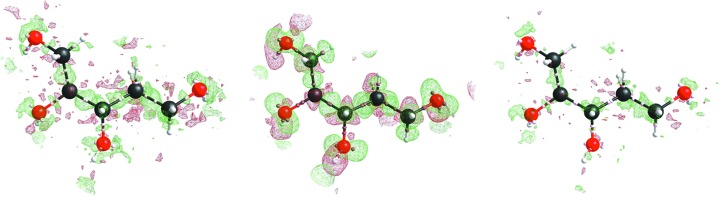
Left: xylitol (sh5011) residual IAM density at ISO level 0.1 e Å^−3^. Center: BODD deformation density added to the IAM. Right: remaining residual density after adding the BODD deformations. IAM 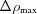/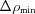 = 0.33/−0.20; BODD 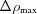/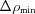 = 0.23/−0.22.

Next the residual density maps after BODD refinement were compared with the conventional IAM residual density maps. Figs. 3 ▸ ▸ ▸ to 6 ▸ show that the BODD model is indeed able to fit most of the aspherical EDD, since the features are significantly reduced in comparison with the IAM residuals.

**Figure 6:**
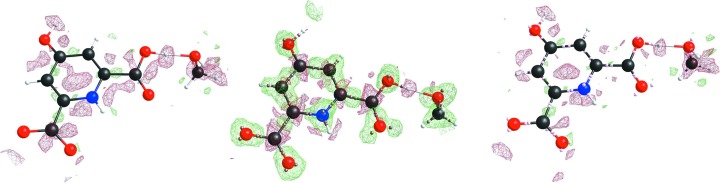
Left: chelidamic acid methanol solvate (eg3095) residual IAM density at ISO level 0.12 e Å^−3^. Center: BODD deformation density added to the IAM. Right: remaining residual density after adding the BODD deformations. IAM 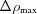/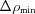 = 0.38/−0.24; BODD 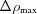/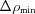 = 0.13/−0.06.

Studying the residual electron-density maps of compound eg3095 (Fig. 6 ▸) reveals that even in cases where the residual density of the IAM model is noisy, and hence apparently does not contain information on bonding electron density, applying the BODD model does not introduce errors (recognizable as new features). Although the reduction of bias by modeling aspherical EDD is smaller for this example, an improvement is still seen. This shows that the BODD model is robust and can safely be used even in the absence of obvious bonding residual density features.

Figs. 3 ▸ ▸ ▸ to 6 ▸ also show the deformation density (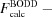 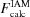), demonstrating that the modeled density is in agreement with a chemist’s expectations. All figures show electron-density maps which were generated with the program *SHELXLE* (Hübschle *et al.*, 2011 ▸).

**Figure 7:**
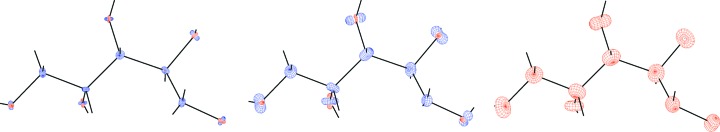
Left: xylitol (sh5011) *Peanut* plot of IAM minus BODD RMSD with a magnification factor of 10. Center: similar IAM minus invariom *Peanut* plot for comparison. Right: BODD minus invariom *Peanut* plot.

While statistical analysis (Table 3 ▸) quantitatively shows that ADPs from BODD and invariom refinement are more similar than both are to the IAM ADPs, this can be more easily conceived visually through *Peanut* plots. Both BODD and invariom model ADPs are systematically smaller than those from the IAM as indicated by the red (smaller) RMSD surfaces in Figs. 7 ▸ ▸ ▸ to 10 ▸. For xylitol, extinction again affects the ADPs, but overall the results are fully consistent with earlier invariom results (Dittrich *et al.*, 2007 ▸).

## 4. Discussion   

All aspherical atom approaches, the new BODD model, SHC databases and Hirshfeld atom refinement alike, have strengths and weaknesses. Least-squares refinement with the SHC multipole model is nearly as fast as the BODD implementation in *SHELXL*, and a rather accurate atomic scattering factor can be stored by using up to 27 parameters, in addition to information on a local atomic coordinate system and an atom type. However, the possibility of having a wrong local coordinate system in cases where an unambiguous definition with SHC scattering factor databases is impossible sometimes requires manual checking and intervention. This problem is solved in BODD, where the local coordinate system is directly deduced from neighboring atoms without user intervention. The relative orientation of BODD with respect to the unit-cell axes is thus hidden from the user.

In comparison with database approaches, Hirshfeld atom refinement can be slightly more accurate – depending on the basis set used – than database scattering factors and is more rigorously defined in terms of the physical–chemical model. It can also include most of the crystal field effect.^15^ Combining the variational principle for single-point energy minimization with least-squares refinement *via* experimental intensities to convergence in turn, Hirshfeld atom refinement is clearly attractive, but also very time-consuming, as it entails considerable computing efforts which increase to the power of four with the number of basis functions. Moreover, Hirshfeld atom refinement cannot handle polymeric structures, and although it can, in principle, be used for disordered structures, the computational effort then increases further. In contrast, disorder can be handled in the BODD model and also in invariom refinement (Dittrich *et al.*, 2016 ▸). The most attractive point for Hirshfeld atom refinement is that it can be combined with X-ray wavefunction fitting (Jayatilaka, 1998 ▸), and this combination is called X-ray wavefunction refinement (Grabowsky *et al.*, 2012 ▸). Both form part of a new research area called quantum crystallography, where recent efforts clearly have the potential to rejuvenate charge-density research (Genoni *et al.*, 2018 ▸) in general. The BODD model has a different focus: user-friendliness and speed, and we again emphasize that the BODD model is not intended to replace detailed studies of the electron density, which require or benefit from using more sophisticated approaches. The BODD model describes the deformation density intuitively using the familiar concepts of bonding electrons and lone pairs rather than the less intuitive multipoles.

## 5. Conclusion   

The BODD model to implement aspherical scattering factors in *SHELXL* provides deformation density in addition to the IAM, is fast, backwards compatible and a user-friendly alternative to database approaches based on the SHC multipole model or to Hirshfeld atom refinement. The pre-computed values of the new BODD database can be directly used *via* the *APEX3* interface and no further computations are required. Scattering factor assignment is nearly fully automatic; when an ideal scattering factor cannot be found in the database suitable alternatives are identified. The model permits the combination of conventional IAM and aspherical scattering contributions, *e.g.* for refinement of a metal complex where no aspherical scattering factor for the metal atom is needed or available.

## Figures and Tables

**Figure 4:**
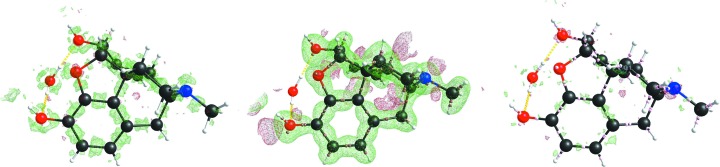
Left: morphine (lc5024) residual IAM density at ISO level 0.2 e Å^−3^. Center: BODD deformation density added to the IAM. Right: remaining residual density after adding the BODD deformations. IAM 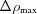/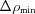 = 0.59/−0.35; BODD 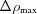/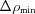 = 0.33/−0.29.

**Figure 5:**
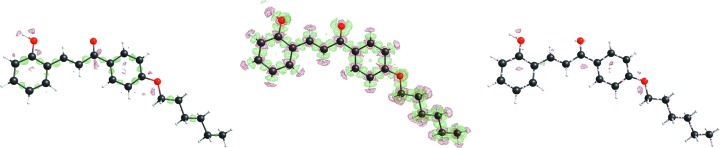
Left: (*E*)-1-[4-(hexyloxy)phenyl]-3-(2-hydroxy-phenyl)prop-2-en-1-one (hb6948) residual IAM density at ISO level 0.08 e Å^−3^. Center: BODD deformation density added to the IAM. Right: remaining residual density after adding the BODD deformations. IAM 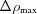/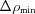 = 0.22/−0.27; BODD 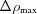/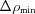 = 0.05/−0.05.

**Figure 8:**
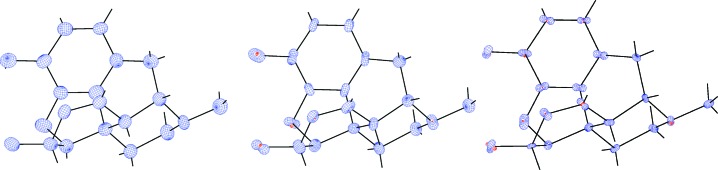
Left: morphine (lc5024) *Peanut* plot of IAM minus BODD RMSD with a magnification factor of 10. Center: similar IAM minus invariom *Peanut* plot for comparison. Right: BODD minus invariom *Peanut* plot.

**Figure 9:**
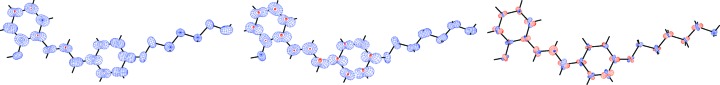
Left: (*E*)-1-[4-(hexyloxy)phenyl]-3-(2-hydroxy-phenyl)prop-2-en-1-one (hb6948) *Peanut* plot of IAM minus BODD RMSD with a magnification factor of 10. Center: similar IAM minus invariom *Peanut* plot for comparison. Right: BODD minus invariom *Peanut* plot.

**Figure 10:**
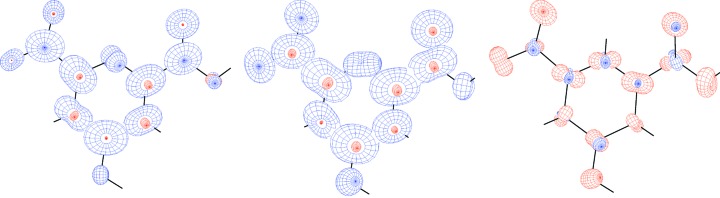
Left: chelidamic acid methanol solvate (eg3095) *Peanut* plot of IAM minus BODD RMSD with a magnification factor of 10. Center: similar IAM minus invariom *Peanut* plot for comparison. Right: BODD minus invariom *Peanut* plot. Only the main molecule is shown.

**Figure 11:**
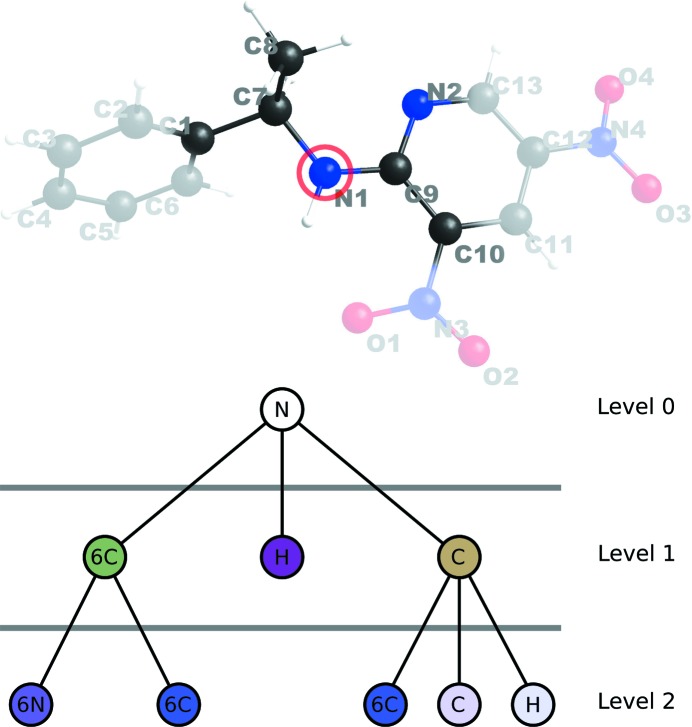
Top: the marked atom’s environment graph is determined. Atoms not contributing to the atom’s environment graph are displayed with reduced opacity. Bottom: environment graph corresponding to atom N1.

**Table 1:** *R*_1_ values before and after applying the BODD model for several structure models The six-character abbreviation is the IUCr publication code.

Compound name	IUCr code	Literature	*R*_1_ (IAM)	*R*_1_ (BODD)
Xylitol	sh5011	Madsen *et al.* (2004 ▸)	2.34	2.07
Morphine	lc5024	Scheins *et al.* (2005 ▸)	3.33	2.56
(*E*)-1-[4-(Hexyloxy)phenyl]-3-(2-hydroxy-phenyl)prop-2-en-1-one	hb6948	Fadzillah *et al.* (2012 ▸)	4.06	3.38
Chelidamic acid methanol solvate	eg3095	Tutughamiarso *et al.* (2012 ▸)	3.66	3.21
Baicalein nicotinamide (1/1)	fg3251	Sowa *et al.* (2012 ▸)	5.80	5.62
2-(1,4,7,10-Tetraazacyclododecan-1-yl)cyclohexan-1-ol (cycyclen)	dt3014	de Sousa *et al.* (2012 ▸)	4.50	4.29
2-(1*H*-Indol-3-yl)-2-oxoacetamide	yp3017	Sonar *et al.* (2012 ▸)	6.35	6.08
*cis*-2-(4-Fluorophenyl)-3a,4,5,6,7,7a-hexahydroisoindoline-1,3-dione	fg3250	Smith & Wermuth (2012 ▸)	3.51	3.22
5-Ethynyl-2′-deoxycytidine	fg3269	Seela *et al.* (2012 ▸)	3.02	2.12
*N*,*N*-Dibenzyl-*N*′-(furan-2-carbonyl)thiourea	fa3263	Pérez *et al.* (2012 ▸)	4.51	4.22
3-Phenylcoumarin	zj2091	Matos *et al.* (2012 ▸)	3.97	3.58
3,4,6-Tri-*O*-acetyl-1,2-*O*-[1-(exoethoxy)ethylidene]-β-D-*manno*-pyranose·0.11H_2_O	bi3042	Liu *et al.* (2012 ▸)	4.84	4.69
4-[(*E*)-(4-Ethoxyphenyl)iminomethyl]phenol	bt5991	Khalaji *et al.* (2012 ▸)	2.98	2.49
*N*,*N*′-Bis(2-methylphenyl)-2,2′-thiodibenzamide	fg3262	Helliwell *et al.* (2012 ▸)	3.55	3.23

**Table 2:** Refinement details of corresponding IAM and BODD refinements

Compound	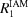	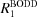	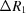	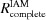	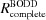	*b*	Resolution (Å)
sh5011	2.34	2.07	0.27	2.77	2.44	0.03	0.41
lc5024	3.33	2.56	0.77	4.49	3.75	0.08	0.44
hb6948	4.06	3.38	0.68	5.31	4.54	0.04	0.73
eg3095	3.66	3.21	0.45	4.61	4.12	0.02	0.82

**Table 3:** Mean difference (MD) in Å and σ(MD) (both ×10^−4^) of corresponding IAM and BODD refinements using invariom refinement as a reference model with the number of values analyzed Values of the overall scale factors (OSF) are also given.

Compound	MD INV-IAM(σ)	MD INV-BODD(σ)	No. of values	OSF IAM	OSF BODD
sh5011	−1.5 (3)	−1.2 (2)	60	1.21	1.21
lc5024	−2.3 (3)	−1.2 (2)	132	0.73	0.70
hb6948	−7.4 (17)	−0.2 (9)	144	1.00	1.01
eg3095	−7.6 (24)	−2.0 (12)	90	1.00	0.97

**Table 4:** Definition of node colors for each node level

Level	Color	Definition
0	r	0
	g	Sum of bond lengths to bonding partners
	b	0
	a	1
1	r	Distance to root atom
	g	Sum of bond lengths to bonding partners
	b	1
	a	1
2	r	Distance to root atom
	g	0
	b	2
	a	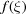

**Table 5:** Row content of the environmental archetype data table

Column	Definition
Environmental graph	The environmental graph is serialized and stored
Inner hash	Inner hash attribute of the graph object
Invariom name	Invariom name generated for the root atom

## Footnotes

1 There has been debate about improving the earlier parameterization to avoid the constant term *c* (Rez *et al.*, 1994 ▸) and improved scattering factors using six Gaussian functions have been tabulated (Su & Coppens, 1997 ▸).

2 This branch of crystallography is called charge density, because the experiment yields not only the electron-density distribution but also the distribution of the atomic nuclei as ADPs.

3 Deformation electron density is the difference between the Fourier transforms of the observed and the calculated structure factors, assuming identical ADPs and phases of the model.

4 Brill (1960 ▸) was one of the first to include bonding electron density in a model for X-ray diffraction data. He showed the improvement of a ‘reliability factor’ upon locating half an electron at the midpoint between bonded C atoms in the model of diamond. Hellner’s work using spherical scatterers should also be mentioned (Hellner, 1977 ▸), since it was more recently re-discovered for modeling protein data (Afonine *et al.*, 2007 ▸, 2004 ▸). There is ongoing research in these developments (Dadda *et al.*, 2012 ▸; Nassour *et al.*, 2014 ▸).

5 This holds true although small perturbations due to the crystal field (Dittrich *et al.*, 2012 ▸) exist. The approximation of using gas-phase molecular density to model the EDD in the solid state is thus entirely appropriate.

6 Other authors prefer the more general term ‘TAAM’ for transferable aspherical atom model (Bąk *et al.*, 2011 ▸), which applies to all current scattering factor databases.

7 We emphasize the DFT-based ‘generalized invariom database’ GID (Dittrich *et al.*, 2006 ▸, 2013 ▸) and the ‘University at Buffalo Databank’ UBDB2011 (Dominiak *et al.*, 2007 ▸; Jarzembska & Dominiak, 2012 ▸). Earlier developments, the ‘supramolecular synthon based fragments approach’ SBFA (Hathwar *et al.*, 2011 ▸) and the ‘experimental library multipolar atom model’ ELMAM2 (Zarychta *et al.*, 2007 ▸; Domagała *et al.*, 2012 ▸), rely on averaged parameters from high-resolution XRD experiments.

8 For an earlier article using this philosophy see Dittrich *et al.* (2005 ▸).

9 A possible solution is a single-point energy computation to provide tailor-made scattering factors (Dittrich *et al.*, 2015 ▸).

10 For the same reason angles between H atoms in methyl groups should be smaller than 109.5°.

11 For aromatic systems at least *A* of LONE 6 will often refine to a negative value. The reason for the negative value lies in the combination of BEDE and LONE instructions applied for modeling of the double bond. The elliptic ED when looking along the bond is obtained by a comparably high *A* for BEDE along the bond, minus four negative amplitudes for BEDE in the negative bond direction of the bonds next to the double one. The latter BEDE functions subtract the ED in the nodal plane of the π-orbital. Hence, LONE 6 just corrects the density orthogonal to that plane directly above and below the atom, where IAM and BEDE place too much EDD.

12 Both invarioms and Hirshfeld atom refinement therefore improve the *R* factor slightly better than do BEDE and LONE. Whether this additional accuracy is indeed required depends on the research question, but the easier implementation of the new model in comparison with the SHC or a quantum-chemical model is a considerable advantage. All models certainly improve conventional structure determination.

13 These features are called IDEAL in the Bruker *APEX3* software.

14 A few of these structures were refined with invarioms earlier; for a more detailed comparison with earlier results see Dittrich *et al.* (2013 ▸) and the respective supplementary information can be consulted.

15 Including the crystal field effect (Dittrich *et al.*, 2012 ▸), *e.g.* by continuous solvent models or by using periodic DFT computations initiated with real crystal structures, is possible, but would be considerably more effort.

## Appendix A Details of technical implementation

### A1. Transferability of similar chemical environments

Chemical equivalence of molecular fragments is used in various ways. A relevant application in the current context is the transfer of aspherical X-ray scattering factors from the above-mentioned databases to experimental structure refinements. Here the selection of the best scattering factor from the database requires that chemical equivalency or chemical similarity can be defined and determined.

The invariom model, which is used as a starting point for this method, parameterizes a chemical environment of an atom in a molecule by generating a sequence of characters encoding the chemical elements and the bonding situation of the atom’s bonding partners – the invariom name. When two atoms give rise to the same invariom name they are considered to be chemically equivalent. While this method has been successfully applied in numerous cases with software provided earlier (Hübschle *et al.*, 2007 ▸), it has its limitations: the generation of invariom names depends on pre-defined thresholds. That implies that two ‘almost equal’ chemical environments can lead to very different invariom names, because one name-determining parameter value is slightly above a threshold in one environment, but slightly below in another. In such cases, the current invariom name-matching method (Dittrich *et al.*, 2005 ▸) will not be able to determine that these two environments are actually very similar. This problem can be mitigated to some degree by using multiple sets of thresholds (Wandtke *et al.*, 2016 ▸). This modification has increased the robustness of the method but cannot solve this problem altogether.

This led to the development of a new similarity-determining protocol that does not depend on equivalence of character strings, but instead is able to quantify similarity of chemical environments with a scalar value. This implies that the new method does not tell if two environments are equal – as the invariom name-matching methods would – but it instead determines how big the difference between two environments is, which will be described next.

### A2. Environment similarity

The proposed method to quantify similarity of chemical environments is based on the abstraction of chemical environments as graph data structures called *environmental graphs*. In this context, a graph is a data structure consisting of connected nodes. A chemical environment of an atom is defined by a root node () that is connected to an additional node () for each bonding partner *i* that particular atom has. Each node  is then connected to one additional node () for each of its corresponding atom’s bonding partners *j* (Fig. 11 ▸).

Each node is then assigned a series of four attributes (*r*, *g*, *b*, *a*) that further define the chemical environment. The ensemble of these four parameters is called a node’s color. Additionally, each node is assigned the parameter *e*, representing the corresponding atom’s elemental symbol. If an atom is part of a planar ring system, the number of ring-atom members is added to the elemental symbol for each ring of which the atom is a part. For example, a C atom that is part of two different planar six-membered ring systems is assigned the symbol 66C.

A node’s color is defined differently for each of the three levels of nodes. Table 4 ▸ lists the definitions. Equations (7) and (8) show the computation of *a* for nodes of level 2,

where  is the ξ value representing a perfect mesomeric bond and  is an empirically determined scale factor,

Here *n* is the level 1 node to which the level 2 node is bonded, *m* is the level 0 node, and  is the bond distance between the corresponding atoms. This means that the *a* attribute is effectively a weighting factor that determines how important a certain node is for determining similarity. Since the *a* value is one for level 0 and level 1 nodes, these nodes are always weighted fully. When it comes to next-nearest neighbors – represented by level 2 nodes – the importance depends on the bond type involving the nearest neighbor and the central atom. If it is an ideal mesomeric bond, the weighting factor is 1. The more it differs from a mesomeric bond, the less weight the next-nearest neighbor gets. The reasoning behind this is that mesomeric bonds can vary significantly depending on the wider chemical surrounding, because the involved molecular orbitals are delocalized across a larger area. Increasing the weight of next-nearest neighbors is an attempt to take that into account. The increased accuracy of this approach has been shown using the invariom model (Holstein *et al.*, 2012 ▸).

The similarity (or distance ) of two environments is then defined as the sum of the pairwise distances () of all their nodes. Since there are several ways to create pairs from the nodes of two environments, all corresponding distances  have to be computed.  is then the permutation of pairs that result in the smallest distance value. Equations (9) and (10) define the computation of :

The contribution  is used as a safeguard to indicate if two nodes of different levels are matched. The contribution  adds a penalty when nodes with different elemental symbols are matched. The value 2 for a mismatch is empirically chosen to result in high  values for mismatches in level 0 and level 1 nodes, but values small enough for the weighting factors  and  compensate for uncritical mis­matches.

Using the color-matching algorithm solves not only the problem of scattering factor assignment in nearly identical chemical environments, it also provides a solution for finding a suitable scattering factor when the best chemical environment is not present in the database. This means that the quantum-chemical computation to provide the best possible scattering factor has not been carried out. Rather than not providing a solution at all, which was the procedure so far, the algorithm will provide a tentative scattering factor that matches the chemical environment better than the IAM would, and the authors of the invariom database^16^ (Dittrich *et al.*, 2013 ▸) can then optionally be informed about the missing chemical environment, which will then be added on a regular basis depending on how often a particular chemical environment is missing.

### A3. Optimization of the color-matching algorithm

The difference-computation protocol can be very costly if a large number of environments are compared with each other. Most atoms in a molecule are bonded to three or four other atoms, resulting in a large amount of permutations to be checked. Therefore, preliminary checks can be implemented to reduce the required amount of comparisons.

When looking for similar environments, the first optimization is to make sure that only those environments are compared that share the same element symbols (including ring membership codes).^17^ This is implemented by generating a string of characters for each environment. The string starts with the element symbol of the root node . Next, a list of the element symbols of all level 1 nodes  is generated and sorted alphanumerically. The resulting list is then concatenated to a character string, where each element is separated by a hyphen. This string is then appended to the root node’s string. This string is named *inner hash*. Now, before performing costly distance computations, the inner hashes of both environments can be compared. In cases where large numbers of environments are stored in a database, the inner hash can be used to query potentially similar environments.

### A4. Mapping chemical environments to tabulated parameters

Determining the similarity between two chemical environments is rarely useful by itself. Instead, it is a powerful tool to access data that are characteristic for certain chemical environments in a robust way. Assuming that it is known how X-rays are scattered by a C atom that is bonded to four other C atoms, and that the equivalent information is known for a C atom bonded to three other C atoms, a chemical similarity search can be used to determine which of these two idealized C atoms is more similar to a C atom found in a structure under study. This is useful, because it is unfeasible to store information about all possible chemical environments. Reducing the whole parameter space to idealized environmental *archetypes* helps to solve this problem.

### A5. Environmental archetypes

An environmental archetype is an idealization of a chemical environment. The virtually infinite and continuous range of possible chemical environments is grouped into discrete archetypes. One such archetype might be the formaldehyde molecule, which defines the parameters of all O atoms connected to a C atom with a double bond. Even though many molecules exist that include O atoms with a double bond to a C atom, it is assumed that these O atoms are similar to the O atom in formaldehyde. In the context of this method, the invariom model is used to define discrete environmental archetypes. The presented method is used to determine which archetype is most similar to any given chemical environment by determining to which archetype an atom in a certain chemical environment belongs.

### A6. Generating an archetype library

The first step in determining an atom’s environmental archetype is the definition of a set of archetypes. The basis for this step was again the invariom database. The library contains thousands of differently bonded atoms of the elements most commonly found in organic chemistry. For each of these atoms an invariom name was determined. Several atoms will generate the same invariom name. In contrast to the invariom model, where rules exist to determine which of these similar environments define a unique invariom name (from which follows which model compound is used to derive a scattering factor), all atoms were used for the archetype library. For each atom its corresponding environmental graph was generated as well. Subsequently, a row is added to a database table containing the information listed in Table 5 ▸.

Some invariom names occur rather often in the invariom database. Some occur several thousand times with very few differences between them. To reduce the size of the database table, and to keep computer applications responsive, the table is optimized using the following algorithm:

If a new row is added to the table and the table contains fewer than 25 rows with the same invariom name, the row is added normally.

If a new row is added to the table and the table contains 25 rows with the same invariom name, the following steps are performed:

For each of the environmental graphs in the 25 rows plus the newly generated row, compute the mean-squared distance  to the 25 other graphs. The graph with the smallest mean-squared difference is the graph most similar to the other graph instances, and likely the most ideal graph representing the given archetype. This graph is kept in the database and is called .

The goal is to keep the database table as diverse as possible. Therefore  between each graph and  are computed. The graph with the smallest distance to  is then removed from the database.

This procedure increases the database-generation time significantly, and solves the issue that upon querying a certain inner hash value thousands of graph instances need to be re-instantiated and compared with a given graph instance. For the more common inner hash values (*e.g.* 6C-6c-6c-h) still about a hundred graphs are stored in the database and provide robust archetype determination.

### A7. Determining the archetype of an arbitrary environment

With the archetype library available, the archetype of arbitrary atoms can be determined efficiently. First, the environment graph of the atom of interest  must be generated. The inner hash value of that graph is then used as a key to query all data table rows containing that hash, yielding a set of graphs . In a next step, the graph distance  is determined for each database entry *i*. The list of graphs  is then sorted by the value of , starting with the smallest. The first element of that list is then the most similar graph, and the invariom name it is linked to represents the archetype of the atom used to generate . For interactive applications it is possible to present the first few elements of the sorted list to the user and allow them to pick the best fit based on chemical knowledge.

Now, the environmental archetype of the atom of interest – defined by an invariom name – is known and data associated with that archetype can be transferred.

## References

[bb1] Afonine, P. V., Grosse-Kunstleve, R. W., Adams, P. D., Lunin, V. Y. & Urzhumtsev, A. (2007). *Acta Cryst.* D**63**, 1194–1197. 10.1107/S0907444907046148 PMC2808317 18007035

[bb2] Afonine, P. V., Lunin, V. Y., Muzet, N. & Urzhumtsev, A. (2004). *Acta Cryst.* D**60**, 260–274. 10.1107/S0907444903026209 14747702

[bb3] Arnberg, L., Hovmöller, S. & Westman, S. (1979). *Acta Cryst.* A**35**, 497–499.

[bb4] Bąk, J. M., Domagała, S., Hübschle, C., Jelsch, C., Dittrich, B. & Dominiak, P. M. (2011). *Acta Cryst.* A**67**, 141–153. 10.1107/S0108767310049731 21325717

[bb5] Brill, R. (1960). *Acta Cryst.* **13**, 275–276.

[bb6] Brock, C. P., Dunitz, J. D. & Hirshfeld, F. L. (1991). *Acta Cryst.* B**47**, 789–797.

[bb7] Capelli, S. C., Bürgi, H.-B., Dittrich, B., Grabowsky, S. & Jayatilaka, D. (2014). *IUCrJ*, **1**, 361–379. 10.1107/S2052252514014845 PMC4174878 25295177

[bb8] Coppens, P. (1967). *Science*, **158**, 1577–1579. 10.1126/science.158.3808.1577 17816628

[bb9] Coppens, P., Sabine, T. M., Delaplane, G. & Ibers, J. A. (1969). *Acta Cryst.* B**25**, 2451–2458.

[bb10] Cruickshank, D. W. J. (1970). *Crystallographic Computing*, edited by F. R. Ahmed, pp. 187–197. Copenhagen: Munksgaard.

[bb11] Dadda, N., Nassour, A., Guillot, B., Benali-Cherif, N. & Jelsch, C. (2012). *Acta Cryst.* A**68**, 452–463.

[bb12] Dauter, Z. (2003). *Methods Enzymol.* **368**, 288–337. 10.1016/S0076-6879(03)68016-X 14674280

[bb13] Debye, P. (1915). *Ann. Phys.* **351**, 809–823.

[bb14] Dittrich, B., Hübschle, C. B., Luger, P. & Spackman, M. A. (2006). *Acta Cryst.* D**62**, 1325–1335. 10.1107/S090744490602899X 17057335

[bb15] Dittrich, B., Hübschle, C. B., Messerschmidt, M., Kalinowski, R., Girnt, D. & Luger, P. (2005). *Acta Cryst.* A**61**, 314–320. 10.1107/S0108767305005039 15846034

[bb16] Dittrich, B., Hübschle, C. B., Pröpper, K., Dietrich, F., Stolper, T. & Holstein, J. J. (2013). *Acta Cryst.* B**69**, 91–104. 10.1107/S2052519213002285 23719696

[bb17] Dittrich, B., Koritsánszky, T. & Luger, P. (2004). *Angew. Chem. Int. Ed.* **43**, 2718–2721. 10.1002/anie.200353596 18629999

[bb18] Dittrich, B., Lübben, J., Mebs, S., Wagner, A., Luger, P. & Flaig, R. (2017). *Chem. Eur. J.* **23**, 4605–4614. 10.1002/chem.201604705 PMC5434951 28295691

[bb19] Dittrich, B., Munshi, P. & Spackman, M. A. (2007). *Acta Cryst.* B**63**, 505–509. 10.1107/S0108768107014838 17507764

[bb20] Dittrich, B., Schürmann, C. & Hübschle, C. B. (2016). *Z. Kristallogr.* **231**, 725–736.

[bb21] Dittrich, B., Sze, E., Holstein, J. J., Hübschle, C. B. & Jayatilaka, D. (2012). *Acta Cryst.* A**68**, 435–442.

[bb22] Dittrich, B., Wandtke, C. M., Meents, A., Pröpper, K., Mondal, K. C., Samuel, P. P., Amin Sk, N., Singh, A. P., Roesky, H. W. & Sidhu, N. (2015). *ChemPhysChem*, **16**, 412–419. 10.1002/cphc.201402600 25393218

[bb23] Domagała, S., Fournier, B., Liebschner, D., Guillot, B. & Jelsch, C. (2012). *Acta Cryst.* A**68**, 337–351. 10.1107/S0108767312008197 22514066

[bb24] Dominiak, P. M., Volkov, A., Li, X., Messerschmidt, M. & Coppens, P. (2007). *J. Chem. Theory Comput.* **3**, 232–247. 10.1021/ct6001994 26627168

[bb25] Doyle, P. A. & Turner, P. S. (1968). *Acta Cryst.* A**24**, 390–397.

[bb26] Fadzillah, S. M. H., Ngaini, Z., Hussain, H., Razak, I. A. & Asik, S. I. J. (2012). *Acta Cryst.* E**68**, o2909. 10.1107/S1600536812038007 PMC3470257 23125701

[bb29] Genoni, A. *et al.* (2018). *Chem. Eur. J.* **24**, 10881–10905.

[bb27] Gillespie, R. J. (1963). *J. Chem. Educ.* **40**, 295–301.

[bb28] Gillespie, R. J. (1970). *J. Chem. Educ.* **47**, 18–23.

[bb30] Grabowsky, S., Genoni, A. & Bürgi, H.-B. (2017). *Chem. Sci.* **8**, 4159–4176. 10.1039/c6sc05504d PMC5576428 28878872

[bb31] Grabowsky, S., Luger, P., Buschmann, J., Schneider, T., Schirmeister, T., Sobolev, A. N. & Jayatilaka, D. (2012). *Angew. Chem. Int. Ed.* **51**, 6776–6779. 10.1002/anie.201200745 22644673

[bb33] Groom, C. R., Bruno, I. J., Lightfoot, M. P. & Ward, S. C. (2016). *Acta Cryst.* B**72**, 171–179. 10.1107/S2052520616003954 PMC4822653 27048719

[bb34] Hansen, N. K. (1978). *MOLLY* – *a computer program for multipole charge-density refinement*. Technical report. Université Henri Poincare, Nancy I, France.

[bb35] Hansen, N. K. & Coppens, P. (1978). *Acta Cryst.* A**34**, 909–921.

[bb36] Hathwar, V. R., Thakur, T. S., Row, T. N. G. & Desiraju, G. R. (2011). *Cryst. Growth Des.* **11**, 616–623.

[bb37] Helliwell, M., Moosun, S., Bhowon, M. G., Jhaumeer-Laulloo, S. & Joule, J. A. (2012). *Acta Cryst.* C**68**, o387–o391. 10.1107/S0108270112035962 23007539

[bb38] Hellner, E. (1977). *Acta Cryst.* B**33**, 3813–3816.

[bb39] Herbst-Irmer, R. & Sheldrick, G. M. (1998). *Acta Cryst.* B**54**, 443–449.

[bb40] Hirshfeld, F. L. (1971). *Acta Cryst.* B**27**, 769–781.

[bb41] Hirshfeld, F. L. & Rabinovich, D. (1973). *Acta Cryst.* A**29**, 510–513.

[bb42] Holstein, J. J., Hübschle, C. B. & Dittrich, B. (2012). *CrystEngComm*, **14**, 2520–2531.

[bb43] Hübschle, C. B., Luger, P. & Dittrich, B. (2007). *J. Appl. Cryst.* **40**, 623–627.

[bb44] Hübschle, C. B., Sheldrick, G. M. & Dittrich, B. (2011). *J. Appl. Cryst.* **44**, 1281–1284. 10.1107/S0021889811043202 PMC3246833 22477785

[bb45] Hummel, W., Hauser, J. & Bürgi, H.-B. (1990). *J. Mol. Graph.* **8**, 214–220. 10.1016/0263-7855(90)80006-2 2282361

[bb46] Jarzembska, K. N. & Dominiak, P. M. (2012). *Acta Cryst.* A**68**, 139–147. 10.1107/S0108767311042176 22186290

[bb47] Jayatilaka, D. (1998). *Phys. Rev. Lett.* **80**, 798–801.

[bb48] Jayatilaka, D. & Dittrich, B. (2008). *Acta Cryst.* A**64**, 383–393. 10.1107/S0108767308005709 18421128

[bb49] Jelsch, C., Guillot, B., Lagoutte, A. & Lecomte, C. (2005). *J. Appl. Cryst.* **38**, 38–54.

[bb50] Jelsch, C., Pichon-Pesme, V., Lecomte, C. & Aubry, A. (1998). *Acta Cryst.* D**54**, 1306–1318. 10.1107/s0907444998004466 10089507

[bb51] Khalaji, A. D., Fejfarová, K. & Dušek, M. (2012). *Acta Cryst.* E**68**, o2646. 10.1107/S1600536812034253 PMC3435674 22969545

[bb52] Koritsánszky, T. S. & Coppens, P. (2001). *Chem. Rev.* **101**, 1583–1627. 10.1021/cr990112c 11709993

[bb53] Liu, Y.-L., Zou, P., Wu, H., Xie, M.-H. & Luo, S.-N. (2012). *Acta Cryst.* C**68**, o338–o340. 10.1107/S0108270112032076 22935499

[bb55] Lübben, J. & Grüne, T. (2015). *Proc. Natl Acad. Sci. USA*, **112**, 8999–9003. 10.1073/pnas.1502136112 PMC4517205 26150515

[bb54] Lübben, J., Volkmann, C., Grabowsky, S., Edwards, A., Morgenroth, W., Fabbiani, F. P. A., Sheldrick, G. M. & Dittrich, B. (2014). *Acta Cryst.* A**70**, 309–316. 10.1107/S2053273314010626 PMC4075069 25970187

[bb57] Madsen, A. Ø. & Hoser, A. A. (2015). *Acta Cryst.* A**71**, 169–174. 10.1107/S2053273314025133 25727865

[bb56] Madsen, A. Ø., Sørensen, H. O., Flensburg, C., Stewart, R. F. & Larsen, S. (2004). *Acta Cryst.* A**60**, 550–561. 10.1107/S0108767304018306 15507737

[bb58] Malaspina, L. A., Edwards, A. J., Woinska, M., Jayatilaka, D., Turner, M. J., Price, J. R., Herbst-Irmer, R., Sugimoto, K., Nishibori, E. & Grabowsky, S. (2017). *CrystEngComm*, **17**, 3812–3815.

[bb59] Malaspina, L. A., White, A. H., Wege, D., Tolmie, M. B., Skelton, B. W. & Grabowsky, S. (2017). *Struct. Chem.* **28**, 1343–1357.

[bb60] Massa, L., Huang, L. & Karle, J. (1995). *Int. J. Quantum Chem.* **56**, 371–384.

[bb61] Matos, M. J., Santana, L. & Uriarte, E. (2012). *Acta Cryst.* E**68**, o2645. 10.1107/S1600536812034277 PMC3435673 22969544

[bb62] Müller, P., Herbst-Irmer, R., Spek, A., Schneider, T. & Sawaya, M. (2006). *Crystal Structure Refinement: a Crystallographer’s Guide to SHELXL*, 1st ed. New York: Oxford University Press.

[bb63] Murshudov, G. (2016). *The Resolution Revolution: Recent Advances in CryoEM*, edited by R. A. Crowther, *Methods in Enzymology*, Vol. 579, pp. 277–305. Elsevier.

[bb64] Nassour, A., Kubicki, M., Wright, J., Borowiak, T., Dutkiewicz, G., Lecomte, C. & Jelsch, C. (2014). *Acta Cryst.* B**70**, 197–211. 10.1107/S2052520613031375 24675589

[bb65] Pérez, H., Corrêa, R. S., Plutín, A. M., O’Reilly, B. & Andrade, M. B. (2012). *Acta Cryst.* C**68**, o19–o22. 10.1107/S0108270111052620 22223282

[bb66] Petříček, V., Dušek, M. & Palatinus, L. (2014). *Z. Kristallogr.* **229**, 345–352.

[bb67] Prince, E. (2004). Editor. *International Tables for X-ray Crystallography*, Vol. C, *Mathematical, Physical and Chemical Tables*, 3rd ed. Dordrecht: Kluwer Academic Publishers.

[bb68] Rez, D., Rez, P. & Grant, I. (1994). *Acta Cryst.* A**50**, 481–497.

[bb69] Rollett, J. S. (1970). *Crystallographic Computing*, edited by F. R. Ahmed, pp. 167–181. Copenhagen: Munksgaard.

[bb70] Scheins, S., Messerschmidt, M. & Luger, P. (2005). *Acta Cryst.* B**61**, 443–448. 10.1107/S010876810501637X 16041094

[bb71] Seela, F., Mei, H., Xiong, H., Budow, S., Eickmeier, H. & Reuter, H. (2012). *Acta Cryst.* C**68**, o395–o398. 10.1107/S0108270112038267 23007541

[bb72] Sheldrick, G. M. (1990). *Acta Cryst.* A**46**, 467–473.

[bb73] Sheldrick, G. M. (2008). *Acta Cryst.* A**64**, 112–122. 10.1107/S0108767307043930 18156677

[bb74] Sheldrick, G. M. (2015). *Acta Cryst.* C**71**, 3–8.

[bb75] Sheldrick, G. M. & Schneider, T. R. (1997). *Methods Enzymol.* **277**, 319–343. 18488315

[bb76] Smith, G. & Wermuth, U. D. (2012). *Acta Cryst.* C**68**, o253–o256. 10.1107/S010827011202447X 22763691

[bb77] Sonar, V. N., Parkin, S. & Crooks, P. A. (2012). *Acta Cryst.* C**68**, o405–o407. 10.1107/S0108270112038322 PMC4028885 23007543

[bb78] Sousa, A. S. de, Sannasy, D., Fernandes, M. A. & Marques, H. M. (2012). *Acta Cryst.* C**68**, o383–o386. 10.1107/S0108270112037195 23007538

[bb79] Sowa, M., Ślepokura, K. & Matczak-Jon, E. (2012). *Acta Cryst.* C**68**, o262–o265. 10.1107/S0108270112024456 22763693

[bb80] Stash, A. I. & Tsirelson, V. G. (2014). *J. Appl. Cryst.* **47**, 2086–2089.

[bb81] Stewart, R. F. (1976). *Acta Cryst.* A**32**, 565–574.

[bb82] Stewart, R. F., Bentley, J. & Goodman, B. (1975). *J. Chem. Phys.* **63**, 3786–3793.

[bb83] Stewart, R. F., Spackman, M. A. & Flensburg, C. (1998). *VALRAY98*. Users Manual. Carnegie Mellon University and University of Copenhagen, Pittsburgh, USA and Denmark.

[bb84] Su, Z. & Coppens, P. (1997). *Acta Cryst.* A**53**, 749–762.

[bb85] Thorn, A., Dittrich, B. & Sheldrick, G. M. (2012). *Acta Cryst.* A**68**, 448–451.

[bb86] Trueblood, K. N., Bürgi, H.-B., Burzlaff, H., Dunitz, J. D., Gramaccioli, C. M., Schulz, H. H., Shmueli, U. & Abrahams, S. C. (1996). *Acta Cryst.* A**52**, 770–781.

[bb87] Tsirelson, V. (2018). *J. Comput. Chem.* **39**, 1029–1037. 10.1002/jcc.24893 28791717

[bb88] Tutughamiarso, M., Pisternick, T. & Egert, E. (2012). *Acta Cryst.* C**68**, o344–o350. 10.1107/S0108270112031691 22935501

[bb89] Volkov, A., Macchi, P., Farrugia, L. J., Gatti, C., Mallinson, P., Richter, T. & Koritánszky, T. (2006). *XD2006 – a Computer Program Package for Multipole Refinement, Topological Analysis of Charge Densities and Evaluation of Intermolecular Energies from Experimental or Theoretical Structure Factors*.

[bb90] Volkov, A., Messerschmidt, M. & Coppens, P. (2007). *Acta Cryst.* D**63**, 160–170. 10.1107/S0907444906044453 17242509

[bb91] Wandtke, C. M., Lübben, J. & Dittrich, B. (2016). *Chem. Phys. Chem.* **17**, 2238–2246. 10.1002/cphc.201600213 26999276

[bb92] Wandtke, C. M., Weil, M., Simpson, J. & Dittrich, B. (2017). *Acta Cryst.* B**73**, 794–804. 10.1107/S2052520617010745 PMC5628397 28980983

[bb32] Woińska, M., Jayatilaka, D., Spackman, M. A., Edwards, A. J., Dominiak, P. M., Woźniak, K., Nishibori, E., Sugimoto, K. & Grabowsky, S. (2014). *Acta Cryst.* A**70**, 483–498. 10.1107/S2053273314012443 25176996

[bb93] Zarychta, B., Pichon-Pesme, V., Guillot, B., Lecomte, C. & Jelsch, C. (2007). *Acta Cryst.* A**63**, 108–125. 10.1107/S0108767306053748 17301471

